# Vitamin D receptor gene polymorphisms in relation to the risk of colorectal cancer in the Polish population

**DOI:** 10.1007/s13277-014-2554-0

**Published:** 2014-09-07

**Authors:** Izabela Laczmanska, Lukasz Laczmanski, Marek Bebenek, Pawel Karpinski, Halina Czemarmazowicz, David Ramsey, Andrzej Milewicz, Maria M. Sasiadek

**Affiliations:** 1 Genetics Department, Wroclaw Medical University, Wroclaw, Poland; 2 Department of Endocrinology and Diabetology, Wroclaw Medical University, Pasteura 4, Wroclaw, Poland; 3 First Department of Surgical Oncology, Lower Silesian Oncology Center, Wroclaw, Poland; 4 Department of Computer Science and Management, Wroclaw University of Technology, Wroclaw, Poland

**Keywords:** Colorectal cancer, Vitamin D receptor, Gene polymorphisms

## Abstract

The protective effect of vitamin D against several cancers including colorectal cancer is modulated by the vitamin D receptor (VDR) and its ligand, the active form of vitamin D. VDR response has been found to play a role in various genes encoding proteins involved in crucial cellular pathways. Single nucleotide polymorphisms (SNPs) of the VDR gene that modulate its activity are located in the promoter region, exons 2–9, and their vicinity and also in the 3′UTR region. Some of them have been previously studied in relation to cancer susceptibility and prognosis. The aim of our study was to investigate four polymorphisms, *Bsm*I, Apa*I*, *Taq*I, and *Fok*I, of the *VDR* gene in Polish patients with sporadic colorectal cancer and to evaluate their association with susceptibility to cancer. We found a significant association between the *Bsm*I genotype and cancer (individuals with the bb genotype are more susceptible to cancer compared to those with other genotypes, *p* = 0.025, Fisher’s exact test for 2 × 2 table). Also, the TT genotype at *Taq*I and the AA genotype at Apa*I* are correlated with a higher risk of cancer (*p* = 0.00071 and *p* = 1.0 × 10^−5^, respectively). We found relatively strong linkage disequilibrium between the *Taq*I and Apa*I* loci (T with A and t with a, respectively). Both of these loci are associated with cancer. We do not observe any such association for the *Fok*I polymorphism. In conclusion, a small modification in VDR expression may play a role in such a multipathway process as tumorigenesis.

## Introduction

Vitamin D is described to have a protective effect against several cancers including colorectal cancer, which is one of the most common cancers in the world [1]. This effect is modulated by the vitamin D receptor (VDR) and its ligand, the active form of vitamin D—1α,25-(OH)_2_-dihydroxyvitamin D (1,25(OH)_2_D_3,_ calcitriol). 1,25(OH)_2_D_3_ is produced locally in many tissues and is the most active natural metabolite of vitamin D [2]. VDR, an intracellular hormone receptor, is expressed in normal colon epithelial cells and other colon cells at various levels, and higher expression correlates with epithelial differentiation and better prognosis, while VDR downregulation is associated with poor prognosis and cancer progression [3]. VDR is a transcriptional regulatory factor that interacts with particular nucleotide sequences of target genes and is encoded by a gene of over 100 kb with two promoter regions, eight coding exons (from 2 to 9), and six untranslated exons (1a–1f) [4]. VDR response plays a role in various genes encoding proteins that act in, e.g., cellular growth, apoptosis, differentiation, or metastasis [2]. Over 60 single nucleotide polymorphisms (SNPs) of the VDR gene located in the promoter region, in exons 2–9, and their vicinity, as well as in the 3′UTR region, that modulate its activity have been described and some of them have been studied in relation to cancer susceptibility and prognosis [1, 2]. A few of them are functional and affect expression of the VDR gene. These include the *Fok*I (rs10735810) polymorphism located in the start codon, the major allele of which produces a protein shorter by three amino acids, and the *Bsm*I (rs1544410), Apa*I* (rs7975232), and *TaqI* (rs731236) polymorphisms located in the 3′UTR region, all involved in regulating the stability of VDR mRNA [5]. The presence of the BAt haplotype (B allele of *Bsm*I, A allele of Apa*I*, and t allele of *TaqI*) is associated with increased expression of VDR [5–7]. SNPs of the VDR gene can influence the level, activity, and properties of the gene transcription of proteins and therefore the biological functioning of vitamin D [8].

The relationship between polymorphisms of the VDR gene and sporadic colorectal cancer has been previously studied [4, 8]. Meta-analysis of a large number of studies revealed that the bb genotype at the *Bsm*I polymorphism of the VDR gene is associated with an increased risk of colon cancer, particularly in the Asian population, although no association with other polymorphisms has been reported [4, 8].

The aim of our study was to investigate four polymorphisms, *Bsm*I, Apa*I*, *Taq*I, and *Fok*I, of the *VDR* gene in Polish patients with sporadic colorectal cancer and to evaluate their association with the risk of cancer.

## Materials and methods

One hundred seventy-nine sporadic colorectal cancers (CRCs) obtained after surgery from the First Department of Surgical Oncology at the Lower Silesian Oncology Centre in Wroclaw were molecularly characterized, including analysis of the *BRAF* V600E (exon 15) and *K-ras* (codon 12 and codon 13) mutations, methylator phenotype (CIMP), and chromosomal instability [9]. The mean age of patients was 65.7 with a standard deviation of 11.2; the number of women was 73 (40.78 %) and the number of men was 106 (59.22 %). All of them were diagnosed with adenocarcinoma coli. Biological material was collected before chemo- and radiotherapy. All the patients were interviewed in regard to their family history of cancer, and those with hereditary cancer syndromes were excluded from the study group. One hundred eighty healthy individuals from the control group were matched according to sex and age.

The study design was accepted by the Ethical Committee of Wroclaw Medical University.

The analysis of the VDR gene polymorphisms, Apa*I*, *Bsm*I, *Fok*I, and *Taq*I, was determined using SNaPshot reaction according to the producer’s protocol (SNaPshot Multiplex Kit, Applied Biosystems) [10] preceded by PCR with a 10x buffer containing 15 mM MgCl_2_, 2 μl (Qiagen); dNTP (2 mM each, Fermentas), 2 μl; *Taq*I polymerase (5 U/μl, Qiagen), 0.3 μl; primers (10 mM), 1 μl; and H_2_O, 7.7 μl to a final volume of 18 μl. These primer sequences are described by Lins et al. [11].

The following PCR and SNaPshot conditions were previously described [12].

Products were separated using an ABI 310 Genetic Analyser with GeneScan Analysis version 3.1.2 software (Applied Biosystems), POP-4 Polymer (Applied Biosystems), and the LIZ 120 size standard (Applied Biosystems) for 15 min. Analysis of the results was performed using GeneMarker version 1.85 software (SoftGenetics LLC).

## Statistical analysis

The chi-squared goodness of fit test was used to investigate the existence of linkage disequilibrium between the rs10735810 (*Fok*I), rs1544410 (*Bsm*I), rs7975232 (Apa*I*), and rs731236 (*Taq*I) *VDR* polymorphisms and whether the genotype frequencies were in agreement with the Hardy-Weinberg equilibrium. When analyzing linkage disequilibrium, an iterative gene counting procedure was used to estimate the haplotype frequencies [13]. Fisher’s exact test was used to analyze the association between the genotypes are these loci and colorectal cancer. The CHAID procedure for hierarchical analysis of these associations was also applied [14].

## Results

The patients were aged between 32 and 87, with a mean age of 65.7 and a standard deviation of 112. It should be noted that there are significantly more males in the study group than females (binomial test, *p* = 0.017), which may indicate that males are more susceptible to colorectal cancer.

The distribution of the genotypes at the Apa*I* and *Taq*I polymorphisms is consistent with the H-W law in the control group (Apa*I* *p* = 0.1675, *Taq*I *p* = 0.1344), but not in the CRC group (Apa*I* *p* = 0.0085, *Taq*I *p* = 0.0230). The *Fok*I polymorphism does not show a significant deviation from the Hardy-Weinberg equilibrium in either the control of CRC group (*p* = 0.8534 and *p* = 0.5616, respectively). The distribution of *Bsm*I genotypes shows a very strong deviation from the Hardy-Weinberg equilibrium, even in the control group (*p* < 10^−5^, see Table 1). We do not observe any association between the *Fok*I polymorphism and colorectal cancer, and there is no linkage disequilibrium with the other loci. There is relatively strong linkage disequilibrium between the *Taq*I and Apa*I* loci in both the study and control groups. The T allele at *Taq*I is associated with the A allele at Apa*I*. The t allele at *Taq*I is associated with the a allele at Apa*I*. Both of these loci are associated with cancer. Those with the TT genotype at *Taq*I and the AA genotype at Apa*I* are more susceptible to cancer than those with other genotypes (*p* = 0.0071 and *p* = 10 × 10^−5^, respectively, Fisher’s exact test for 2 × 2 table). There is a significant association between the *Bsm*I genotype and cancer. Those with the bb genotype are more susceptible to cancer, *p* = 0.025 (see also Table 2).

**Table 1:** Hardy-Weinberg equilibrium

Polymorphism	Genotype	Observed	Expected	Chi-square	*p* value
Control group					
rs731236 (*Taq*I)	TT	57 (31.9 %)	52.0 (29.1 %)	2.24	0.1344
	Tt	79 (44.1 %)	89.0 (49.7 %)		
	tt	43 (24.1 %)	38.0 (21.2 %)		
rs7975232 (Apa*I*)	AA	29 (15.4 %)	33.6 (17.9 %)	1.9	0.1675
	Aa	101 (53.7 %)	91.8 (48.8 %)		
	aa	58 (30.9 %)	62.6 (33.3 %)		
rs1544410 (*Bsm*I)	BB	86 (45.7 %)	71.6 (38.1 %)	**19.82**	0.0000*
	Bb	60 (31.9 %)	88.9 (47.3 %)		
	bb	42 (22.3 %)	27.6 (14.7 %)		
rs10735810 (*Fok*I)	FF	62 (33.0 %)	62.6 (33.3 %)	0.03	0.8534
	Ff	93 (49.5 %)	91.8 (48.8 %)		
	ff	33 (17.6 %)	33.6 (17.9 %)		
Study group					
rs731236 (*Taq*I)	TT	74 (47.2 %)	67.6 (43.1 %)	**5.17**	0.0230*
	Tt	58 (36.9 %)	70.9 (45.1 %)		
	tt	25 (15.9 %)	18.6 (11.8 %)		
rs7975232 (Apa*I*)	AA	58 (37.4 %)	50.0 (32.2 %)	**6.92**	0.0085*
	Aa	60 (38.7 %)	76.0 (49.1 %)		
	aa	37 (23.9 %)	29.0 (18.7 %)		
rs1544410 (*Bsm*I)	BB	38 (41.3 %)	24.0 (26.1 %)	**34.07**	0.0000*
	Bb	18 (19.6 %)	46.0 (50.0 %)		
	bb	36 (39.1 %)	22.0 (23.9 %)		
rs10735810 (*Fok*I)	FF	47 (28.7 %)	48.8 (29.8 %)	0.34	0.5616
	Ff	85 (51.8 %)	81.3 (49.6 %)		
	ff	32 (19.5 %)	33.8 (20.6 %)		

Numbers in brackets give % value

*Significant difference from Hardy-Weinberg equilibrium

**Table 2:** Frequencies of genotypes of VDR polymorphisms in sporadic CRC

Polymorphism	Genotype	CRC	Control	Chi-square	*p* value^d^	OR	95 % CI
rs731236 (*Taq*I)	TT	74 (47 %)	57 (33 %)	7.655	0.0220	1.84^a^	1.15–2.95
	Tt	58 (37 %)	78 (44 %)				
	tt	25 (16 %)	40 (23 %)				
rs7975232 (Apa*I*)	AA	58 (37 %)	29 (16 %)	20.479	0.0000	3.14^b^	1.84–5.47
	Aa	60 (39 %)	100 (55 %)				
	aa	37 (24 %)	53 (29 %)				
rs1544410 (*Bsm*I)	BB	38 (41 %)	42 (46 %)	6.316	0.0411	2.13^c^	1.08–4.31
	Bb	18 (20 %)	28 (31 %)				
	bb	36 (39 %)	21 (23 %)				
rs10735810 (*Fok*I)	FF	47 (29 %)	60 (33 %)	0.866	0.6553	n/d	n/d
	Ff	85 (52 %)	91 (50 %)				
	ff	32 (19 %)	31 (17 %)				

*n/d* not determined

^a^OR of TT genotype with respect to other genotypes

^b^OR of AA genotype with respect to other genotypes

^c^OR of bb genotype with respect to other genotypes

^d^Using Fisher’s exact test for association for the 3 × 2 table

## Discussion

There is no doubt that the risk of sporadic colorectal cancer is associated with such environmental factors as dietary habits, lifestyle, and also genetic factors. It has also been revealed that vitamin D intake and the level of 25-hydroxyvitamin D in serum are inversely associated with colorectal cancer [4]. The role of VDR in various cellular pathways suggests it plays a crucial role in the etiology and development of cancer [15]. Many studies on VDR gene polymorphisms and sporadic CRC have shown a reduced risk of CRC for patients with the B allele of *Bsm*I, f allele of *Fok*I and A allele of Apa*I*, and t allele of *Taq*I, which are associated with higher activity of the vitamin D receptor, but the influence of SNPs on cancerogenesis may be modified by many elements including the environment, lifestyle, and many other genetic factors [2, 8, 16]. In our study, we found that the following genotypes are associated with a higher risk of colorectal cancer: the bb (CC) genotype at the *Bsm*I locus, the TT (AA) genotype at *Taq*I, and the AA (GG) genotype at Apa*I*. There is no statistical evidence for any association with *Fok*I (5′-end polymorphism). We observed relatively strong linkage disequilibrium between the *Taq*I and Apa*I* loci in both the study and control groups. The T allele at *Taq*I is associated with the A allele at Apa*I*. The t allele at *Taq*I is associated with the a allele at Apa*I*. When the association of the Apa*I* genotype to cancer is taken into account, there is no significant association between the *Taq*I genotype and cancer. However, when the association of the *Taq*I genotype to cancer is taken into account, the association between the Apa*I* genotype remains significant. This suggests that the Apa*I* genotype is more important in modulating an individual’s susceptibility to cancer, and the association between the *Taq*I genotype and cancer may result from the linkage disequilibrium between *Taq*I and Apa*I*.

We also found a significant association between the *Bsm*I genotype and cancer. Those with the bb genotype are more susceptible to cancer. However, the association between the *Bsm*I locus and the *Taq*I locus is very odd. In the control group, there is linkage disequilibrium of medium strength between *Taq*I and *Bsm*I. In the study group, the degree of linkage disequilibrium is large. The data indicate that among those whose *Bsm*I genotype is bb (high risk), those who also have genotype TT at *Taq*I are clearly more susceptible to cancer. However, among those whose *Bsm*I genotype is not bb (lower risk), those who have genotype TT at *Taq*I are clearly less susceptible to cancer.

Results from various meta-analyses have clearly shown the statistically significant association of the B allele of *Bsm*I with reduced sporadic CRC risk using both a dominant model (BB + Bb versus bb) and recessive model (BB versus Bb + bb) [2, 8]. Our results are in agreement with other studies considering *Bsm*I, which suggest that the B allele of the *Bsm*I polymorphism has a protective effect against cancerogenesis in a variety of ethnical populations [8]. The *Bsm*I polymorphism does not modify the amino acid sequence of proteins, mRNA, or protein levels. However, it exhibits strong linkage disequilibrium with other VDR SNPs, including Apa*I* and *Taq*I. This suggests that the *Bsm*I, Apa*I*, and *Taq*I polymorphisms have a significant influence on VDR expression or mRNA processing [17].

Results obtained from many studies and observed in meta-analyses are the most reliable and have the highest statistical value. Numerous studies on different gene polymorphisms have shown that SNPs should be examined and interpreted in functional groups (e.g., groups of polymorphisms that reduce or enhance an enzyme’s activity) [2, 8]. Although the conclusions obtained are sometimes contradictory because of differing features of various populations, ethnic differences, and even differences between groups of patients, the accumulation of data from many published results is statistically more valid and can detect genetic associations with higher accuracy, while reducing the probability of false-negative results [4, 8, 18].

## Conclusion

Even a small modification in VDR activity or ligand affinity may play a crucial role in such a multipathway process as tumorigenesis due to the significant engagement of VDR in biological processes in cells and its role in homeostasis.
